# Towards personalised management of atherosclerosis via computational models in vascular clinics: technology based on patient-specific simulation approach

**DOI:** 10.1049/htl.2013.0040

**Published:** 2014-03-21

**Authors:** Vanessa Díaz-Zuccarini, Giulia Di Tomaso, Obiekezie Agu, Cesar Pichardo-Almarza

**Affiliations:** 1 UCL Mechanical Engineering, University College London, Multiscale Cardiovascular Engineering Group, Torrington Place, London, WC1E 7JE, UK; 2 Vascular Unit, University College Hospital, 235 Euston Road, London, NW1 2BU, UK; 3 InScilico Ltd, London, EC1 V 4PW, UK

**Keywords:** health care, diseases, vascular service unit, computer simulations, national health systems, personalised healthcare, patient-specific simulation approach, vascular clinics, computational models, atherosclerosis, personalised management

## Abstract

The development of a new technology based on patient-specific modelling for personalised healthcare in the case of atherosclerosis is presented. Atherosclerosis is the main cause of death in the world and it has become a burden on clinical services as it manifests itself in many diverse forms, such as coronary artery disease, cerebrovascular disease/stroke and peripheral arterial disease. It is also a multifactorial, chronic and systemic process that lasts for a lifetime, putting enormous financial and clinical pressure on national health systems. In this Letter, the postulate is that the development of new technologies for healthcare using computer simulations can, in the future, be developed as in-silico management and support systems. These new technologies will be based on predictive models (including the integration of observations, theories and predictions across a range of temporal and spatial scales, scientific disciplines, key risk factors and anatomical sub-systems) combined with digital patient data and visualisation tools. Although the problem is extremely complex, a simulation workflow and an exemplar application of this type of technology for clinical use is presented, which is currently being developed by a multidisciplinary team following the requirements and constraints of the Vascular Service Unit at the University College Hospital, London.

## 1 Introduction

Cardiovascular disease (CVD) is the main cause of illness and premature death in the EU, accounting for approximately 40% of deaths. CVD costs the EU over 192 billion euros a year and is estimated to account for more than a quarter of all disability-adjusted life years lost in the EU [1]. CVD is forecast to remain the leading cause of disability in developed countries for many years. Within the wide spectrum of CVD, atherosclerosis has been singled out as the leading cause of death.

Atherosclerosis is a degenerative condition in which an artery wall thickens as the result of a build-up of lipids (primarily cholesterol and fatty acids) and fibrosis of the wall. It is commonly referred to as a ‘hardening’ or ‘furring’ of the arteries. In the early stages, an accumulation of lipid-laden macrophages (foam cells) is seen in the subendothelium. With time, smooth muscle cells and fibrous tissue will accumulate. The formation of lesions is enhanced by plasma proteins carrying elevated levels of cholesterol and triglycerides, and also blood pressure is a driving force of atherosclerosis progression.

Atherosclerosis is a chronic, progressive and multifactorial disease with a long asymptomatic phase. Clinical manifestations of atherosclerosis including coronary artery disease (also called atherosclerotic heart disease), cerebrovascular disease and peripheral arterial disease will occur in 2 of 3 men and 1 in 2 women after the age of 40. Subclinical atherosclerosis is a latent precursor of clinical CVD, including myocardial infarction and stroke [2].

According to the European Society of cardiology [1], patient outcomes could be greatly improved if we were able to shift from treatment to prevention. Now, more than ever, national health systems must deal with the pressures facing governments trying to maintain health provisions under fiscal constraints, with health funders experiencing difficulties in meeting the demands of modern health systems. Pressures arise from many quarters, including: an ageing population needing longer and more intensive medical treatments; population increase and patient and clinician demands for the latest equipment/services, among others. Fundamental understanding of the formation of atherosclerosis, cost-effective management and cost-controlled treatment of the disease in the long term are becoming essential. However, atherosclerosis is a complex process and the progression of the disease will depend on patient-specific anatomical characteristics (which influence the mechanical stimuli on the endothelium), as well as environmental and lifestyle factors (such as age and smoking) and genetic variables. The complicated framework that encapsulates plaque formation and the myriad of cause-effect relationships at multiple levels make management of the condition almost intractable for clinicians who, nevertheless, must deal with it every day. Hence, an in-silico management and support system, based on predictive models (including the integration of observations, theories and predictions across a range of temporal and spatial scales, scientific disciplines, key risk factors and anatomical sub-systems) combined with digital patient data and visualisation tools would offer much needed help.

National and international guidelines provide evidence and recommendations to assist clinicians in managing and treating atherosclerosis [3, 4]. However, evidence is limited on identifying individuals with subclinical atherosclerosis before a CVD event occurs, especially since specialised diagnostic imaging is not part of routine clinical practice [2]. The purpose of this Letter is to present a simulation tool that integrates medical and physiological data, to enable the development of predictive models to understand the evolution and progression of atherosclerosis in individual patients.

In this case, only biomechanical stimuli, patient-specific anatomical data and measurements of individual LDL levels are included in the modelling framework presented here, but the ultimate goal of this work would be to integrate individual patient data at all relevant levels of organisation, from the genome to the organism level, and to include environmental factors to produce personalised predictive models, with the objective of improving patient care. It is important to highlight that the work presented here is the result of an extendable and expansible in-silico framework and could be adapted to future knowledge and discoveries. It is also important to mention that this in-silico framework and the model used as the basis of this Letter do not present a ‘monolithic vision’ but are able to adapt to future requirements instead. This is key, as this work was initiated by the requirements of clinical practitioners at the University College Hospital to begin to address some of the challenges they experience in their routine clinical practice.

This paper is divided as follows: Section 2 presents in a condensed way the key elements of the mathematical model used as the basis of the in-silico workflow. Section 3 presents the simulation workflow. An exemplar application of a patient-specific simulation and its discussion will be presented in Section 4. Finally, Section 5 presents the conclusions of this work.

## 2 Mathematical model

**Figure 1:**
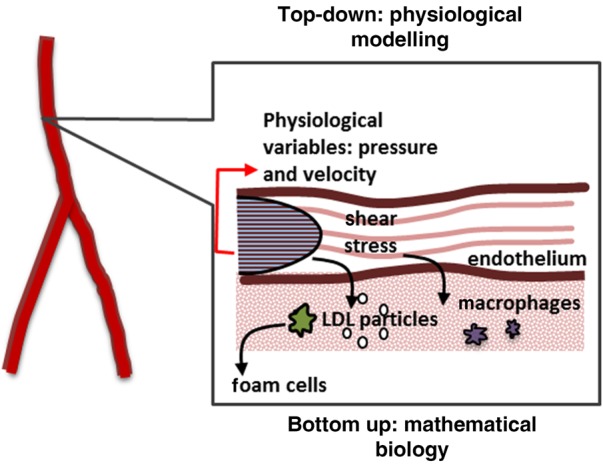
Representation of formation of foam cells and initiation of atherogenesis LDL particles enter arterial wall through endothelium; monocytes are recruited and once in the wall, differentiate into macrophages. These macrophages will capture LDL particles and the result will be foam cells

The cornerstone of the technology presented here is a mathematical model, described below; the reader is also referred to [5] for completeness. The model captures patient-specific and multi-scale aspects of the atherogenesis process as briefly explained in Section 1. The essence of the model is encapsulated in Fig. 1.

### 2.1 Capturing and modelling the hemodynamics

Capturing patient-specific hemodynamics is fundamental as atherosclerotic-prone regions are linked with regions of low shear stress (see Section 2.2). The calculation of physiological variables, such as velocities and shear stress, will provide quantitative information of the mechanical stimuli exerted by the blood flow on the endothelium and the endothelial cells. The hemodynamics of the problem are calculated using the continuity (1) and Navier-Stokes (2) as shown below

(1) $$\displaystyle{{\partial \rho } \over {\partial t}} + \nabla \cdot \lpar \rho \; U\rpar = 0\eqno\lpar 1\rpar $$

(2) $$\displaystyle{{\partial \lpar \rho \; U\rpar } \over {\partial t}} + \nabla \cdot \lpar \rho \; U \times U\rpar = - \nabla p + \nabla \cdot \tau + S_M \eqno\lpar 2\rpar $$

where, *p* and *τ* are the pressure and stress tensor, respectively, [Pa] and *U* is the flow velocity (m/s). The term *S_M_* accounts for the momentum of external forces to the system.

### 2.2 Correlation between endothelium behaviour and wall shear stress – the endothelium as a porous wall

The endothelium is the linen of blood vessels and the interface between the blood flow and the arterial wall (Fig. 1). Its behaviour is heavily influenced by local hemodynamics. *In-vivo* experimental findings show that, in areas of altered hemodynamics, the endothelial cell does not have a typical cobblestone shape [6] showing a more circular shape instead. These more circular shaped endothelial cells show an increased permeability, creating areas of higher macromolecular migration inside the arterial wall. The model uses a relationship between endothelial permeability and local wall shear stress (WSS) based on the endothelial cell shape index (SI) taken after experimental findings from [6]. SI is related to the cellular shape and takes values from zero to one; that is, a circle has a SI of one while a straight line has a SI of zero.

It was assumed that the value of normal WSS for an artery could be calculated using the Poiseuille law for laminar flow in a straight pipe with the artery radius [7].

The relationship between WSS and SI is modelled by a continuous function following [8]:

(3) $$\hbox{SI} = - 0.2435 \cdot \displaystyle{{\tau ^{0.3537} } \over {\tau _0 }} + 0.4025\eqno\lpar 3\rpar $$

where *τ* corresponds to the local WSS value and *τ*_0_ is the normal WSS.

### 2.3 LDL passage through the endothelium and membrane-transport model

To model LDL passage through the endothelium, a modified version of the Kedem Ketchalsky's equations for membrane transport was used [4]:

(4) $$J_v = L_p \left({\Delta p_{{\rm end}} - \sigma _d \Delta \Pi } \right)\eqno\lpar 4\rpar $$

(5) $$J_s = P_i \left({c_{{\rm lum}} - c_{w\comma {\rm end}} } \right)\displaystyle{{{\rm Pe}} \over {e^{{\rm Pe}} - 1}} + J_v \left({1 - \sigma } \right)c_{{\rm lum}} \eqno\lpar 5\rpar $$

(6) $${\rm Pe} = \displaystyle{{J_v \lpar 1 - \sigma \rpar } \over {P_i }}\eqno\lpar 6\rpar $$

where *J_v_* is the volumetric flux through the endothelium, *L_p_* is the hydraulic conductivity, Δ*p*_end_ is the pressure difference through the endothelium, σ_d_ is the osmotic reflection coefficient and ΔΠ is the osmotic pressure (negligible, when compared with the hydraulic pressure through the endothelium) [5].

The solute flux, *J_s_*, can be divided into a convective component entering the membrane and a diffusive component represented by the first and last terms of (5), respectively. *P_i_* is the diffusive permeability, Pe the modified Peclet number (6), *c_w_*_,end_ the LDL concentration in the arterial wall at the sub-endothelial layer and σ the solvent drag coefficient [5].

**Figure 2:**
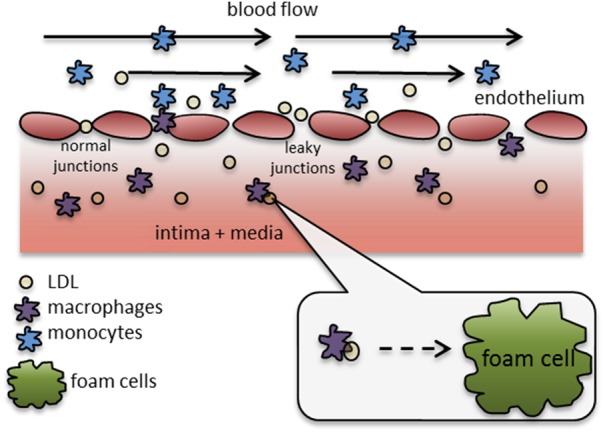
Schematic representation of biochemical processes of plaque formation, key molecules and cells, endothelium junctions and intima + media

Three main pathways of macromolecule penetration were considered: leaky endothelial cell junctions; normal endothelial cell junctions; and vesicular pathways [5]. The model considers that the bulk of volume flux through the endothelial membrane is given by:

(7) $$J_v = J_{v\comma lj + } J_{v\comma nj} \eqno\lpar 7\rpar $$

where *J_v,lj_* is the flux through leaky junctions and *J_v,nj_* is the flux through normal junctions, as indicated in Fig. 2. The arterial wall was modelled using an electrical analogy, where the flow is driven by a pressure difference and the resistance to the flow entering the arterial wall is given by the endothelial layer

(8) $$J_v = \displaystyle{{\Delta p_{{\rm end}} } \over {R_{{\rm end}} }}\eqno\lpar 8\rpar $$

Here, Δ*P*_end_ is the pressure difference through the endothelium, taken from experimental values [9]. *R*_end_ is the resistance of the endothelium and was calculated as the parallel sum of the resistance of leaky junctions and normal junctions [8]

(9) $$\displaystyle{1 \over {R_{{\rm end}} }} = \displaystyle{1 \over {R_{lj} }} + \displaystyle{1 \over {R_{nj} }}\eqno\lpar 9\rpar $$

where *R_lj_* is the resistance through the leaky junctions, calculated as

(10) $$R_{lj} = \displaystyle{1 \over {L_{\,p\comma lj} }}\eqno\lpar 10\rpar $$

and *R_nj_* is the resistance through the normal junctions, calculated as

(11) $$R_{nj} = \displaystyle{1 \over {L_{\,p\comma nj} }}\eqno\lpar 11\rpar $$

To describe the LDL transport through normal junctions, normal endothelial permeability (*P_nj_*) and hydraulic conductivity (*L_p,nj_*) values were taken from experimental results (Table 1).

**Table 1:** LDL transport

Symbol	Quantity	Value
*α*	LDL radius	11 nm
*A_lj_*	leaky cell area	3.775 × 10^−12^ m^2^
*D_c_*	LDL diffusion coefficient in the wall	8 × 10^−13^ m^2^/s
*D_m_*	monocytes diffusion coefficient in the wall	1 × 10^−3^ mm^2^/s
*K*	Darcy's permeability of arterial wall	2.9 × 10^−18^ m^2^
*k_m_*	foam cell formation constant	1 × 10^−6^ s^−1^
*L_p,nj_*	hydraulic conductivity of normal junctions	1.58 × 10^−9^ m(s·mm Hg)
*L_p,lj_*	hydraulic conductivity of single leaky junctions	2.94 × 10^−11^ m/(s·mm Hg)
*P_v_*	diffusive permeability of vesicular pathway	1.92 × 10^−11^ m/s
*P_s,lj_*	diffusive permeability of single leaky junction	2.5 × 10^−6^ m/s
*r_w_*	LDL degradation rate	3 × 10^−4^ s^−1^

Following the three pores theory [8], solute flux does not occur through normal endothelial cell junctions, but it only occurs through endothelial leaky cell junctions and vesicles

(12) $$J_s = J_{s\comma lj} + J_{s\comma v} \eqno\lpar 12\rpar $$

where the solute flux through the vesicular pathway (*J_s,v_*) is calculated as 10% of the solute flux through the leaky junction pathway (*J_s,lj_*) [4]. Having defined the transport properties through both the vesicular pathway and the normal junction pathway, the remaining transport properties to be defined are the ones describing the leaky junction pathway.

Leaky cells have a high permeability to macromolecules such as LDL, a factor that can be linked to the magnitude of WSS on the endothelium. Experimental findings show that, in areas of low WSS and high endothelial SI, the number of mitotic cells (MCs) is increased [10], leading to a relationship between endothelial SI and a number of MCs [8] that can be used to calculate the number and the proportion of leaky cells over normal cells. This would allows to determine the properties of the endothelium such as hydraulic conductivity and diffusive permeability. The equations are not shown here but see [4] for details. The values are shown in Table 1.

### 2.4 Modelling the atherosclerosis process inside the arterial wall – a transport model

A monolayer approach was used, considering the arterial intima and media with the internal elastic lamina and the external elastic lamina as forming one single layer. The transport of LDL inside the arterial wall was modelled in the direction normal to the arterial lumen, via a convection-diffusion-reaction equation [4]

(13) $$\displaystyle{{dc_w } \over {{\rm d}t}} = - {\bi u}_{\bi w} \cdot \nabla c_w + D_w \Delta c_w - r_w c_w \eqno\lpar 13\rpar $$

The transmural velocity ***u_w_*** is calculated using Darcy's law

(14) $${\bi u}_{\bi w} = \displaystyle{K \over {\mu _p }}\nabla p\eqno\lpar 14\rpar $$

where *μ*_p_ is the viscosity of plasma. The transport properties of the arterial wall with respect to LDL are described by Darcy's equation, where the arterial wall has permeability *K* and a diffusion coefficient *D_w_*. The last term of (13) is the degradation of the LDL particles, with *r_w_* as the reaction coefficient.

A relationship between the LDL concentration at the sub-endothelial layer (*c_w_*_,end_) and the adventitia (*c*_w,adv_) of (*c*_w,adv_/*c*_w,end_) = 0.005 (taken from [11]) was used as a boundary condition. Owing to space limitations, see [5] for details on the oxydation model and parameters used.

Once inside the arterial wall it is assumed that all monocytes would differentiate into macrophages. Their transport has been modelled with a diffusion-reaction equation

(15) $$\displaystyle{{dM_w } \over {{\rm d}t}} = D_m \Delta M_w - k_m L_{{\rm ox}} M_w \eqno\lpar 15\rpar $$

Finally, the formation of foam cells (*F_w_*) in the arterial wall is calculated:

(16) $$\displaystyle{{dF_w } \over {{\rm d}t}} = k_m L_{{\rm ox}} M_w \eqno\lpar 16\rpar $$

where *k_m_* is the kinetic constant for foam cells formation.

Foam cells are key in the process of remodelling of the arterial wall and they were considered to be accumulating spheres following close hexagonal packing. To simulate the initial process leading to the plaque formation, it is proposed that, if the volume covered by foam cells was larger than the initial arterial wall portion considered, this would impose a lumen-side narrowing of the arterial wall.

### 2.5 Description of the lumen-free approach

Concentration inside the arterial blood was considered constant for both LDL and monocytes, following a lumen-free approach. It is important to note that the equations shown in this Section represent different phenomena happening at different temporal and length scales.

## 3 Multi-scale in-silico workflow for atherogenesis and the patient-specific simulation of plaque formation

**Figure 3:**
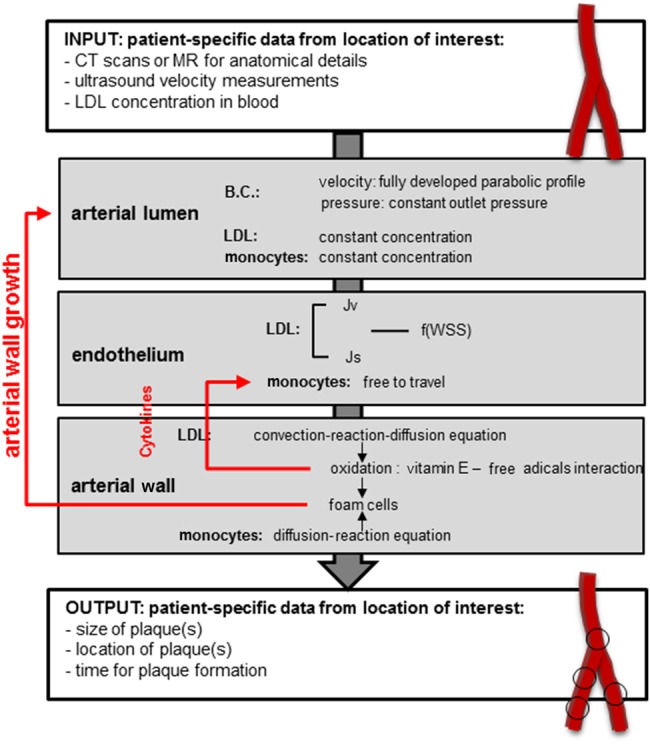
Atherosclerosis remodelling cycle There is a feedback loop between the remodelling of the arterial wall and the calculation of the permeability of the endothelium, which will be affected becoming more porous as atherosclerosis progresses, facilitating the penetration of LDL particles and increasing the number of foam cells. This process is captured in time

Since atherosclerosis formation is a long-timescale process, we will introduce for the purpose of this Letter what we will call an ‘atherosclerosis remodelling cycle’, which defines the whole process of plaque formation and remodelling of the arterial wall. This remodelling involves capturing patient-specific hemodynamics for an individual at a time when the artery under study was considered to be healthy, then the calculation of the intima-media thickening via the set of equations defined in Section 2 and finally the remodelling of the lumen as a consequence of atherosclerosis formation via estimation of the number of foam cells. Fig. 3 illustrates the workflow of the atherosclerosis remodelling cycle.

The core elements of the model are presented in this workflow and these are based on the equations presented in Section 2. It is important to mention that this model is able to simulate, as a whole, very long time scales. The simulation of this long-term process has been rationalised by identifying diverse temporal scales involved as follows: the first and smallest time scale describes the arterial wall model (accumulation of LDL and formation of foam cells). This scale is of the order of hours/days. The second time scale is used to update the endothelium model according to the change of LDL concentration inside the arterial wall. This scale is of the order of days/weeks. The third time scale is the time length of the entire atherosclerosis remodelling cycle. This scale is of the order of months or years.

Please note that the arterial haemodynamics were simulated in steady-state, hence temporal changes in haemodynamic variables are not captured with the current setup. A new haemodynamic simulation was run (and new values of blood flow-related variables calculated) when the growth calculated by the model described in Section 2 was large enough (of the order of μm) to be captured in 3D simulation; meaning, there were small but quantifiable differences in flow characteristics between the ‘previous’ geometry and the ‘new’, calculated one. Once the WSS patterns and values change, the permeability of the endothelium changes, increasing the penetration of macromolecules into the wall creating a feedback loop (see Fig. 3).

This workflow was developed with the aim of producing a comprehensive framework for the multi-scale modelling of atherosclerosis. Different computer tools/software were used: the hemodynamics in the arterial lumen were modelled in Ansys CfX v.14. The arterial endothelium and arterial wall were modelled and implemented in Matlab. The meshing was done in Ansys ICEM CFD. The simulation workflow has been entirely parallelised and we would like to think it presents a valid alternative to the patient-specific modelling and simulation of plaque, as it is separated into individual components and is able to harness the power of parallel computing to perform calculations that would be otherwise intractable, in order to simulate long periods of time.

## 4 Exemplar application of the simulation-based technology– a personalised model of atherogenesis

**Figure 4:**
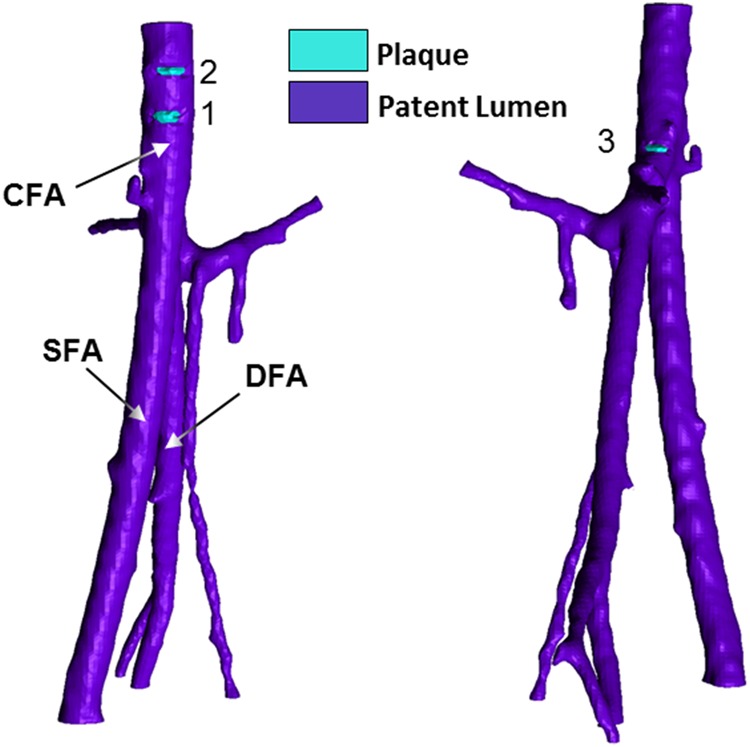
Geometry of model of left CFA, with DFA and SFA and plaque scoring obtained from CT scans Atherosclerotic plaques are segmented in contrast colour (light blue) from the patent lumen (purple): detected plaques are numbered. Note that these are two views of the same arteries in order to show the three plaques detected

Patient-specific data was obtained from the vascular clinic at UCLH. Appropriate patient consent for patient data use was obtained by the managing clinician. The segmented patent lumen geometry of the left common femoral artery (CFA), with the deep femoral artery (DFA) and the superficial femoral artery (SFA) from one of the patients in the study is shown in Fig. 4.

The inlet area of the left CFA is *A*_0, leftCFA_ = 7.84 × 10^−5^ m^2^, with a surface area of the inlet added pipe of *A*_inlet_ = 6.09 × 10^−5^ m^2^. The mean velocity of the left CFA, as extracted from Doppler Echocardiography recordings, was *U*_0_ = 0.207 m/s. Blood was modelled as a steady, homogeneous, incompressible fluid with constant viscosity. Blood properties were kept constant with a viscosity of *μ* = 0.0035 Pa s and a density of *ρ* = 1050 kg/m^3^ [7]. Flow continuity was applied to calculate the mean velocity at the inlet of the pipe. The endothelial response to the local WSS values was tuned with the calculated Poiseuille law WSS for the left CFA. The Poiseuille WSS was calculated to be *τ*_0_ = 0.657 Pa.

Multi-slice computed tomography (MSCT) plaque scoring was carried out with the assistance of a clinician on the arterial segment of the left CFA with the DFA and the SFA, as shown in Fig. 4. The lesions identified were in the left CFA and this is the segment that will be subject to remodelling during the simulation. Owing to the small extent of calcification detectable in the MSCT images, there is uncertainty in the plaques detected and their segmentation. The fluid domain of the left CFA, with the DFA and the SFA, was meshed with Ansys ICEM CFD. The mesh created was a 1.1 million elements Delaunay unstructured mesh with prismatic layers at the wall boundary.

**Figure 5:**
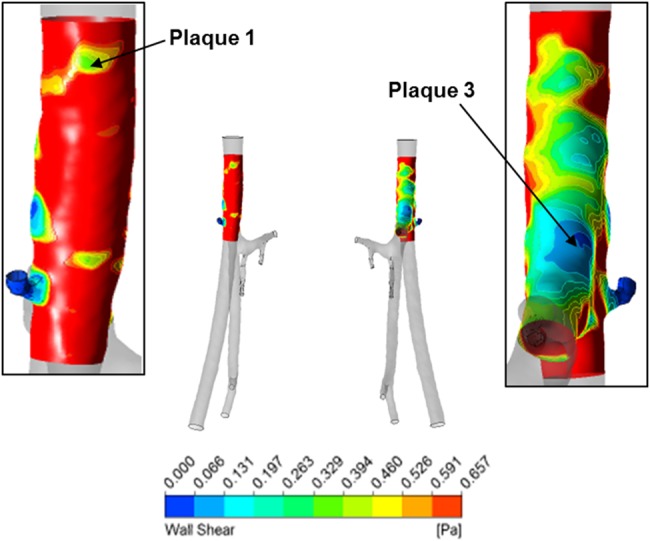
Re-scaled WSS contour plot of left CFA Plot maximum WSS detectable is Poiseuille WSS for left CFA *τ*_0_ = 0.6572 Pa

To obtain the characteristics of the lumen geometry before disease occurrence, the plaques detected were segmented together with the free lumen. An artery-specific WSS contour plot is shown in Fig. 5, obtained by re-scaling the global WSS contour plot, by setting the maximum WSS as the calculated Poiseuille WSS value for a pipe with the same cross sectional dimension and mean flow velocity of the artery considered.

**Figure 6:**
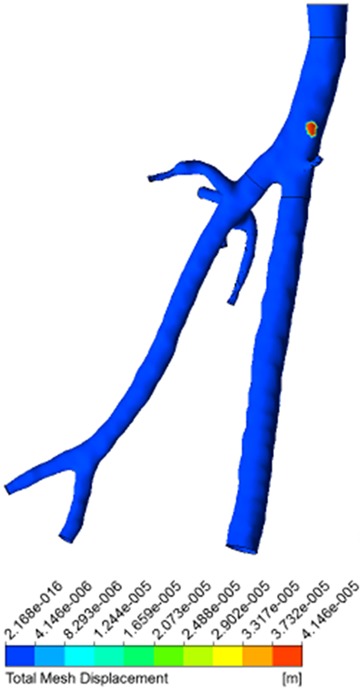
Mesh deformation contour plot of left CFA with DFA and SFA Plaques occurrence was modelled solely in left CFA (in red); area of mesh displacement indicates plaque formation

In this exemplar, two out of the three plaques (1 and 3 in Fig. 4) were shown by the computational model, either in the normalised WSS plot (Fig. 5) or the mesh deformation plot (Fig. 6). Plaque 2 was not developed by the model or shown by the normalised WSS contour plot in the location identified by the figure showing plaque scoring (Fig. 4); however, there is a region of low WSS above plaque 1, which could correspond to plaque 2. It is important to mention that from *in-vivo* observations, it has emerged that the majority of plaques would develop in areas close to arterial bifurcations and this has been already reported numerous times in the literature [12]. It could be, therefore, necessary to segment every different branch, even the minor ones, to accurately model the artery hemodynamics. This fact could affect the generation of plaque 2 by the model.

Additionally, we are using imaging data available routinely through the National Health Service, which in essence means that the model is dealing with data that was not acquired for research purposes. In the case of the exemplar shown here, there is a non-negligible influence of the quality of the images and the successive errors that might have been produced during their processing.

The development of the plaque, shown in Fig. 6, has a simulated time of 20 years and 9 months. Careful consideration must be given to the meaning of the time indication of atherosclerosis formation in this simulation. The anatomical features were extracted from an adult patient and it was assumed that the arterial tree of the patient would reach full anatomical development in 18 years, without presenting any atherosclerosis formation and with arterial lumen and an arterial wall fully patent. This is, however, just an indication of the capabilities of the model to simulate long periods of time. It is only fair to mention that the hypotheses and the model proposed account for processes that happen during the early stages of plaque formation. Other assumptions and mechanisms for the modelling of later stages of the plaque will be considered in future work.

Although the flow was simulated using a rigid wall approximation, the assumption of a rigid wall is not expected to influence the results in this case as a steady state simulation is performed [13].

There is obviously room for improvement in the development of this technology. First of all, it is important to mention that the model, as it is, is not intended to be used for the analysis of plaque rupture. Investigations of the two inter-connected processes (plaque growth and rupture) have been intensive in the past several decades with excellent work available in the literature. Second, it is important to highlight that this is a parsimonious model of atherosclerotic plaque formation. The model captures the fundamental biological hypotheses of plaque formation available in the literature, but it would require the incorporation of many other mechanisms deemed important for the formation of atherosclerosis in individuals. For example, specific genotypes and gene expression (apoE for example) are not considered. It would also be interesting to explore the contribution of other well-known risk factors of atherosclerosis, such as high blood pressure and smoking. One of the challenges in all mechanistic models lies in the correct identification of the relevant or appropriate mechanisms that will result in the expected outcome. This is not an easy obstacle to overcome as we are only able to model current knowledge. However, this model does work as an integrator or hypotheses tester. The fact that this model can efficiently simulate very long time scales (simulating years of growth) in a few days is, per se, an important asset as the atherosclerosis process can then be validated against *in-vivo* data, either in animals or humans (using clinical data if available). Another aspect that requires further improvement is that the model is very sensitive to segmentation and meshing. It is rather difficult to obtain high quality images for all the patients as we are working with data that is routinely available in vascular clinics. Images that are limited in their quality negatively affect the potential of the model and the routine-use of the framework.

To partially overcome these obstacles, a study on a population of patients (with follow-up) at the University College Hospital will be undertaken. This will allow us to collect relevant data to refine the model, to test it, to validate it numerically but also, and very importantly, to validate it in the clinic. The objective is that this study will not only help to develop the technology further but will also help to break the barriers of adoption of this type of technologies in routine clinical practice in the vascular service at UCLH.

## 5 Conclusion

The development of personalised in-silico technologies for better healthcare is key to address the many constraints that National Health systems face today. In this Letter, we presented an in-silico framework to comprehend and manage atherosclerotic plaque progression in individual patients. The fundamental idea behind this technology is that, by understanding the underlying biology and physiology through the encapsulation of this knowledge into mathematical models, we will be able to create rich and persuasive predictive technologies for healthcare. The model-based technology described in this Letter presents an extensible model that can help clinicians to test hypotheses to understand plaque progression in individual patients in order to make more informed decisions about timescales, potential interventions, available treatments and, potentially, lifestyle changes. In the near future, we will work even more closely with clinical colleagues in the vascular service at UCLH to ensure that our research contributes to the broader objectives of the vascular clinic in the hospital and eventually beyond. The ultimate goal is that this framework could become part of routine diagnosis or routine care.
